# Effect of Silver Nanoparticles With Thermoplastic Polyurethane on Postoperative Rehabilitation of Diabetic Patients With Open Fracture of Lower Extremities

**DOI:** 10.3389/fsurg.2022.954155

**Published:** 2022-07-11

**Authors:** Dong Zhang, Dongchen Yao, Ruofei Ma, Shaokui Nan, You Lv, Yue Zhu, Shiwen Zhu

**Affiliations:** ^1^ Department of Orthopedic Surgery, The Sixth Medical Center of PLA General Hospital, Beijing, China; ^2^ Department of Traumatology and Orthopaedics, Beijing Jishuitan Hospital (The Fourth Medical College of Peking University), Beijing, China

**Keywords:** nano-silver, open fracture, diabetes mellitus, rapid rehabilitation, postoperative recovery

## Abstract

**Objective:**

This retrospective study aims to explore the effect of silver nanoparticles with thermoplastic polyurethane (TPU/NS) on the rehabilitation of diabetic patients with open fracture of lower extremities.

**Methods:**

Diabetic patients (*n* = 98) with open fracture of lower extremities treated in our hospital were analyzed retrospectively from June 2015 to December 2021. TPU/NS nanocomposites were prepared for postoperative treatment of diabetic patients with open fracture of lower extremities. First, the cultured *Staphylococcus aureus* and *Escherichia coli* were used to test the antibacterial effect of TPU/NS dressing *in vitro*. After using TPU/NS dressing (observation group) and traditional dressing (control group), the inflammatory reaction, clinical treatment, functional rehabilitation, and adverse reactions in patients were compared.

**Results:**

TPU/NS dressing effectively inhibited the growth of bacteria with a minimum inhibitory concentration of 2 μg/mL. The usage of TPU/NS dressing reduced the inflammatory reaction by reducing positive rate of bacteria after the dressing on the seventh day postoperatively. Besides, the times of dressing, stopping time of wound exudation, wound healing time, length of hospital stay, and VAS score in the observation group were lower than those in the control group; the incidence of adverse reactions after treatment was lower in the observation group as compared with the control group (17.07% vs. 35.09%). Meanwhile, the functional rehabilitation and life quality of patients in the observation group were better TPU/NS dressing treatment.

**Conclusion:**

TPU/NS dressing has the function of promoting the postoperative recovery of patients by inhibiting the bacterial infection of the wound, thus improving the limb function and life quality. As a result, there was a tremendous potential to apply the constructed TPU/NS membrane to diabetic patients with open fractures, especially those with soft tissue injury.

## Introduction

Poorly controlled diabetes mellitus (DM), a common metabolic disease, negatively affects outcomes associated with several lower extremity orthopedic conditions and complications including fractures (1, 2). Moreover, higher baseline fracture risks in both type I (T1MD) and type II diabetes (T2MD) were reported to result in a higher overall incidence of fractures in women (3). There was an expected rise to 366 million individuals with DM by 2030 worldwide; therefore, the incidence of open fractures of the lower limb is also on the rise (3, 4). The wounds have the characteristics of foreign body pollution, serious tissue defects, and poor blood supply, which are easily associated with infection, especially in patients with diabetes (5). If wound infection occurs after fixation surgery, it may lead to delayed healing, multiple debridements, bone and soft tissue defects, and joint stiffness, resulting in permanent loss of function and even amputation (6, 7).

It is generally believed that most of the pathogens causing the infection are introduced during injury or surgery (8). Infection means that there are sufficient numbers of toxic microorganisms, which can destroy the local defense mechanism of patients (9). Once the bacteria invade, any given systemic antibiotics are difficult to play the effective roles (10). Therefore, we are looking for a postfracture wound dressing to effectively prevent infection of fracture wounds.

Nanosilver (NS) as one type of excellent antibacterial agent has strong and broad-spectrum antiviral activity against both Gram-positive and -negative bacteria, including multiple drug tolerance bacteria, for example, methicillin-resistant *Staphylococcus aureus* (MRSA) (11). What is more, there is some proposal that NS suppressed bacteria *via* various mechanisms, such as cell membrane destruction, DNA replication interference, and respiratory function inhibition without causing drug resistance (12). Therefore, NS may be an appropriate antibacterial agent contained in biological materials. However, choosing a suitable wound dressing is still a question that needs to be explored (13). Thermoplastic polyurethane (TPU) is a type of biocompatible and biodegradable elastomer that has been approved by the Food and Drug Administration (FDA) and has been widely used in biomedical sciences (14), which does not only display significant chemical stability but also good mechanical behavior (15). These excellent properties indicate that TPU has the potential to become a kind of wound dressing (16).

In this study, prepared TPU/NS antibacterial wound dressings were applied to the postoperative wounds of open fracture patients with DM to observe and analyze its effect on rehabilitation and pain improvement.

## Materials and Methods

### Clinical Information

The clinical data of patients with open fractures of lower limbs treated in our hospital from June 2015 to December 2021 were retrospectively analyzed. Inclusion criteria are as follows: (1) the clinical symptoms and imaging examination met the diagnostic criteria in *Guidelines on Diagnosis and Treatment of Open Fractures in China (2019 Edition)* (17); (2) DM was confirmed in line with the *Diagnosis and Classification of Diabetes Mellitus published by* American Diabetes Association (18); (3) all patients had new lower limb fractures and met the surgical treatment standards; (4) Gustilo grade: type II and type IIIA; (5) age > 18 years. Exclusion criteria are as follows: (1) patients had malignant tumors and pathological fracture; (2) wound debridement of patients were in grade C; (3) patients had coagulation dysfunction, vascular diseases, and autoimmune diseases; (4) patients had diabetic ulcers; (5) patients had mental disorders, cognitive dysfunction, or Alzheimer’s disease; (6) patients had renal or severe cardiopulmonary dysfunction; (7) patients were allergic to nanosilver; (8) patients had a preoperative infection; and (9) patients whose soft tissues hardly be second-stage sutured even if skin- muscle flap transfer, free skin grafting nor vacuum suction device. A total of 98 patients were included, including 59 males and 39 females. Their average age was 56.22 ± 19.53 years. The patients were divided into an observation group (using TPU/NS dressing, 41 cases) and a control group (using traditional dressing coated with benzalkonium chloride or iodophor, 57 cases) according to propensity score matching (PSM). All patients agreed to participate in the experiment and signed informed consent. This study has been approved by the hospital ethics committee, and it complies with the Declaration of Helsinki.

### Preparation of TPU/NS

TPU membrane (Lubrizol, USA) placed into dopamine (DA) (Sangon, Shanghai, China) solution was soaked at 25°C for 20 h. During this period, membrane color turned from light white into dark brown. Then, the DA-coated film was placed into a 5 mM AgNO_3_ solution at 25°C for avoiding light for 6 h. At last, ion water was used to wash the sample twice followed by stoving at a temperature of 45°C lasting 4 h to obtain TPU/NS dressings. Scanning electron microscopy (SEM, Crossbeam 340, Zeiss, Germany) was adopted to observe the morphology of original TPU and TPU/NS films and energy-dispersive X-ray spectroscopy (EDS) was used to conduct compositional analysis. ImageJ software was adopted to measure silver nanoparticles' average pore size, porosity, and diameter, and two independent researchers read the data.

### Antibacterial Properties of TPU/NS *In Vitro*

*S. aureus* (ATCC6538, Shanghai Huzheng Biotechnology Co., Ltd, China) and *Escherichia coli* (CICC10662, Shanghai Huzheng Biotechnology Co., Ltd) are common Gram-positive and Gram-negative bacteria in nosocomial infection, respectively, which are used to detect antibacterial properties of TPU/NS dressing. *S. aureus* and *E. coli* were cultured in a 24-well plate with bacterial liquid media containing tryptic soy broth (BD Bacto, Becton, Dickinson and Company, USA; pH 7.3) in a microorganism incubator (51028133, Thermo Fisher, USA) at 37°C and 5% CO_2_ according to a previous study (19). The cultured bacteria were adjusted to a concentration of 1 × 10^6^ CFU/ml. The diluents of *S. aureus* and *E. coli* were divided into a blank control group and a TPU/NS group, in which a small number of TPU/NS nanocomposites were added to the TPU/NS group. At the same time, the synthetic TPU/NS solution was diluted into different concentration gradients by the double dilution method, which were added to the Petri dish with bacteria (1 × 10^6^ CFU) for detecting the minimum inhibitory concentration. The activation states of *S. aureus* and *E. coli* were tested by a live/dead bacterial viability kit (AAT-B22411, AAT Bioquest, USA). In this kit, MycoLight 520 solution and propidium iodide (PI) solution were used for viable (green) and nonviable (red) bacteria, respectively.

### Therapeutic Method

After fully wound debridement, appropriate fixation methods were adopted for the fracture, and the wound shall be sutured and disinfected. For the wound that could not be sutured in the first stage, the suture or skin grafting shall be performed after the infection was controlled in the second stage. Antibiotics were given half an hour before the operation, which were used three consecutive days after the operation. The additional usage of antibiotics was based on the wound infection. In addition, all patients were given health education about DM, as well as the customize diabetes diet program by the nutrition department. Meanwhile, daily blood glucose monitoring and medication guidance should be implemented. In terms of postoperative wound dressing, the patients were sutured and disinfected routinely on the open wounds. The patients in the control group were treated with traditional dressings coated with benzalkonium chloride or iodophor, while those in the observation group with TPU/NS dressings. During the dressing on the seventh day postoperatively, the exudates from the wounds of the patients should be extracted for bacterial culture, then the wound shall be disinfected with iodophor, and then, the corresponding dressings were used. The outcome indicators were evaluated 2 months after the operation.

### Inflammatory Response Test

Before treatment and 1, 3, 7, and 10 days after treatment, two tubes of 5 ml of venous blood were taken from each patient, and one tube was centrifuged at 3,000 rpm to obtain serum. Levels of white blood cell (WBC), neutrophils (NEU%), and erythrocyte sedimentation rate (ESR) were measured by an automatic blood cell analyzer (XN-2000, XISEN Meikang), C-reactive protein (CRP) by the automatic biochemical analyzer (AU-680, Beckman), as well as concentrations of interleukin-6 (IL-6) and tumor necrosis factor-α (TNF-α) were detected by an automatic chemiluminescence immunoanalyzer (DXI-800, Beckman). If the above indicators had a downward trend, it was considered an “improved inflammation status.” During the dressing on the seventh day postoperatively, the wound secretion was taken for bacterial culture, and the positive rates of bacterial between the two groups were compared.

### Evaluation of Clinical Treatment and Adverse Reactions

The clinical recovery of patients is evaluated through the times of dressing, stopping time of wound exudation, wound healing time, and length of hospital stay. The visual analog scale (VAS) (20) score is used to evaluate the pain degree of patients’ wounds. The lower the score, the lower the pain. The adverse reactions of the two groups were recorded, including wound infection, delayed healing, scar hyperplasia, soft tissue necrosis, and fracture pain.

### Comparison of Functional Rehabilitation

The functional assessment scale (FIM) (21) was used to evaluate the functional rehabilitation of the two groups of patients, which was divided into self-care function, action, and cognitive function. The higher the score, the better the lower limb function recovery. In terms of life quality, the Orthopedic Quality of Life (SF-36) questionnaire (22) was used to evaluate patients’ quality of life. The questionnaire contained some items such as physical function, emotional function, role function, social function, and other dimensions. Each dimension was expressed by 0–100 points; the higher the score, the better the patients’ quality of life.

### Statistical Methods

SPSS 20.0 (SPSS, Chicago, IL, USA) and GraphPad Prism 8.0 (GraphPad Software, San Diego, California, USA) were used to analyze data and draw the statistical picture. Measurement data presented as mean ± standard deviation (SD) were compared using a *t*-test between two groups, and enumeration data were compared using a *χ*^2^ test. A value of *P* < 0.05 indicated that this difference showed statistical significance.

## Results

### Comparison of General Information of Both Group Patients

The baseline information of the patients from the observation group (*n* = 41) and the control group (*n* = 57) is shown in Table 1. There were no remarkable differences in gender, age, BMI, Gustilo grade, wound location, limb distribution, wound area, FBG, HbA1c, smoking history, history of alcoholism, and cultural level between the two groups (all *P* > 0.05)

**Table 1:** General information table.

Factor	Observation group (*n* = 41)	Control group (*n* = 57)	*χ*2	*P*
Gender			0.082	0.775
Male	24 (58.54%)	35 (61.40%)		
Female	17 (41.46%)	22 (38.60%)		
Age (years)			0.674	0.412
≤55	16 (39.02%)	27 (47.37%)		
>55	25 (60.98%)	30 (52.63%)		
BMI (kg/m^2^)			2.421	0.120
≤23	13 (31.71%)	27 (47.37%)		
>23	28 (68.29%)	30 (52.63%)		
Gustilo grade			0.917	0.338
II	22 (53.66%)	25 (43.86%)		
III A	19 (46.34%)	32 (56.14%)		
Wound location				
Lateral	17	28		
Medial	13	15		
Bilateral	11	14	0.595	0.743
Limb distribution				
Left	20	32		
Right	21	25	0.519	0.471
Smoking history			1.543	0.214
Yes	20 (48.78%)	35 (61.40%)		
No	21 (51.22%)	22 (38.60%)		
History of alcoholism			0.231	0.631
Yes	8 (19.51%)	9 (15.79%)		
No	33 (80.49%)	48 (48.21%)		
Cultural level			2.211	0.137
High school and above	22 (53.66%)	39 (68.42%)		
Below high school	19 (46.34%)	18 (31.58%)		
Wound area (cm^2^)	45.26 ± 5.16	44.77 ± 6.55	0.398	0.691
FBG (mmol/L)	9.82 ± 3.19	9.21 ± 3.02	0.963	0.338
HbA1c (%)	8.19 ± 1.89	8.57 ± 1.71	−1.038	0.302

### Evaluation of Bacteriostasis of TPU/NS Dressing

**Figure 1:**
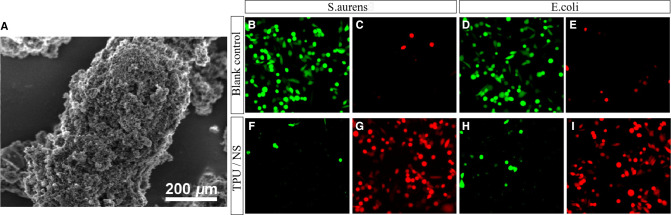
Characterization and antibacterial properties of TPU/NS. (**A**) Scanning electron microscopy image of TPU/NS. (**B,C**) Viable and nonviable staining of cultured *S. aureus* (**B,C**) and *E. coli* (**D,E**) 12 h in the blank control group. (**F**–**I**) Viable and nonviable staining of cultured *S. aureus* (**F,G**) and *E. coli* (**H,I**) 12 h after adding TPU/NS nanocomposites.

Due to the exposure of soft tissue and bone, the wound of open fracture is very easy to be complicated with infection. Effectively killing bacteria and reducing viable bacteria were important methods to promote rehabilitation of patients and prevent related complications. In this study, *S. aureus* and *E. coli* were selected to study the antibacterial effect of TPU/NS dressing. We observed the characteristics of TPU/NS by SEM. The results showed that TPU/NS nanoparticles were evenly distributed and well dispersed, with a spherical shape of 95 nm (Figure 1A). Meanwhile, TPU/NS dressing could reduce viable (green) bacteria with increased nonviable (red) bacteria (Figures 1B–I), and the minimum inhibitory concentration of TPU/NS was 2 μg/ml. The above results show that TPU/NS dressing can effectively use silver ions to achieve excellent antibacterial properties.

### Comparison of Serum Inflammatory Reaction and Positive Detection Rate of Wound Bacteria

**Figure 2:**
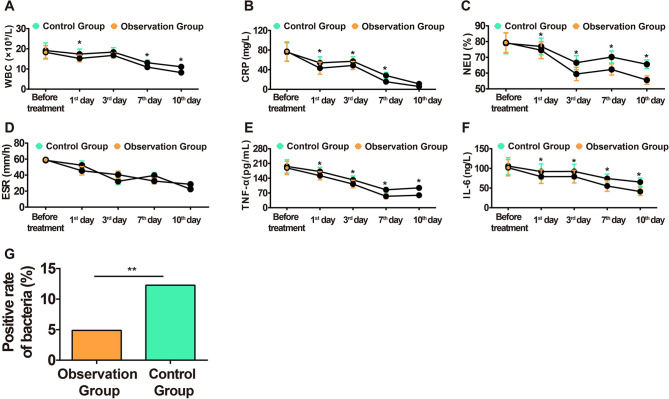
Comparison of inflammatory reaction in both groups. (**A**) Changes in the WBC level in blood. (**B**) Changes in the CRP level in serum. (**C**) Changes in the NEU% level in blood. (**D**) Changes in the ESR level in blood. (**E**) Changes in the TNF-α level in serum. (**F**) Changes in the IL-6 level in serum. (**G**) Bacterial positive detection rate on the 7th day after the operation. *P < 0.05, **P < 0.01.

Inflammatory factors can promote the occurrence of the inflammatory cascade, which is an important indicator of the degree of inflammation. It was found that the TPU/NS dressing could reduce WBC, NEU%, CRP, IL-6, and TNF-α in serum as compared to the control dressing without affecting ESR between both groups (Figures 2A–F). In addition, TPU/NS dressing suppressed the positive detection rate of bacteria (4.88% vs. 12.28%) (Figure 2G). The above results suggested that TPU/NS dressing can better reduce the level of inflammatory factors to prevent the occurrence of infection.

### Comparison of Clinical Treatment

**Figure 3:**
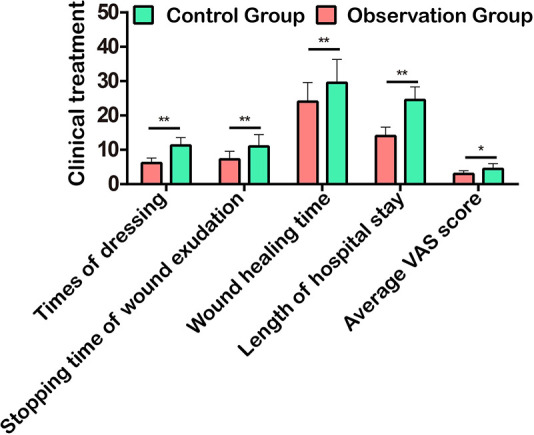
Comparison of clinical treatment in terms of times of dressing, stopping time of wound exudation, wound healing time, hospital stay, and average VAS score. *P < 0.05, **P < 0.01.

The clinical treatment of patients was evaluated from the aspects of the times of dressing, stopping time of wound exudation, wound healing time, hospital stay, and average VAS score. The study showed that the above indexes in the observation group were significantly lower than those in the control group (Figure 3), suggesting that the use of TPU/NS dressing effectively promoted patients’ rehabilitation and reduced the pain of wounds.

### Comparison of Adverse Reactions After Treatment

The incidence of adverse reactions after treatment was evaluated in terms of wound infection, delayed healing, scar hyperplasia, soft tissue necrosis, and fracture pain. The study found that the total incidence of the above adverse reactions in the observation group was significantly lower than that in the control group (17.07% vs. 35.09%, Table 2), suggesting that the use of TPU/NS dressing can better inhibit the proliferation of bacteria and promote wound recovery.

**Table 2:** Comparison of postoperative complications.

Group	Cases	Wound infection	Delayed healing	Scar hyperplasia	Soft tissue necrosis	Pain at the fracture site	Total incidence
Observation group	41	2 (4.88)	2 (4.88)	1 (2.44)	1 (2.44)	1 (2.44)	7 (17.07)
Control group	57	7 (12.28)	5 (8.77)	3 (5.26)	3 (5.26)	2 (3.51)	20 (35.09)
χ2							3.877
*P*							0.049

### Comparison of Functional Rehabilitation and Life Quality

**Figure 4:**
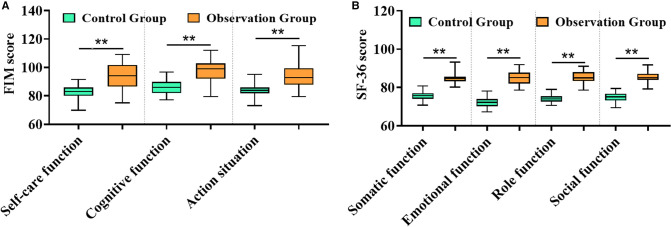
Comparison of rehabilitation between both groups. (**A**) FIM score. (**B**) SF-36 score. **P < 0.01.

After treatment, the recovery indexes of low limb function in self-care function, cognitive function, and action of patients in the observation group were significantly better than those in the control group (Figure 4A), and the physical function, emotional function, role function, social function, and other dimensions of their life quality were remarkably higher than the other (Figure 4B), suggesting that TPU/NS dressing is more conducive to wound recovery to better promote the rehabilitation of patients.

## Discussion

During the treatment of open fractures, patients often had seriously wound due to high-violence injury, and tension blisters and wound infection are easy to appear in the later stage, resulting in the adverse impact on the recovery of postoperative wounds, the long treatment cycle, as well as poor prognosis (23). In addition, during the treatment, the degree of pain in the wound is also a key factor affecting the rehabilitation effect. Although the pain can promote blood circulation in patients, excessive pain was reported to have an adverse impact on postoperative rehabilitation (24). Therefore, taking effective prevention and treatment for infection after the operation has important clinical significance for postoperative recovery and suppression of complications (25).

With the continuous development of nanomedical technology in the past decade, nanodressings have gradually been applied in clinical practice (26). Bacterial infections have long been a thorny issue in clinical anti-infective treatment, and NS as a new type of antibacterial agent has the advantages of high antibacterial activity and a broad antibacterial spectrum without drug resistance (27). Due to the exposure of soft tissue and bone in the wound of open fracture, it is very easy to be complicated with infection. Effectively killing bacteria and reducing viable bacteria are important methods to promote patients’ rehabilitation and prevent related complications. Therefore, NS dressing brings hope to the clinical postoperative anti-infection. The NS dressing using a TPU membrane as a carrier processes pure silver into elemental silver particles with a particle size of about 10 nm and loads them on the TPU membrane. In the antibacterial property experiments, it was found that TPU/NS dressing had a significant antibacterial effect in patients accompanied by the reduced inflammatory reaction, the improved functional rehabilitation, and the alleviated postoperative wound pain. Subsequently, we also tested the positive rate of bacteria in the two groups during the dressing on the seventh day postoperatively. The results showed that the positive rate in the observation group was significantly lower. This also suggests that the application of the nanosilver dressing in open fracture postoperative patients can effectively reduce wound bacteria.

A previous study has pointed out that, unlike the single target effect of antibiotics, NS has a variety of ways to inhibit bacteria (28). For example, it can cause obvious morphological changes in the bacteria by attaching to the surface of bacteria, destroying its cell wall and cell membrane, causing a large number of substances required for maintaining bacterial metabolism to leak, and even causing bacteria to lyse and die. In addition, it has been pointed out in other literature studies that nanosilver can react with enzymes or proteins containing sulfur groups, thus affecting the metabolism of bacteria and at the same time collapsing proton power, leading to the death of bacteria (29).

We compared the times of dressing, stopping time of wound exudation, length of hospital stay, and wound healing time between the two groups, and the results showed that the clinical treatment of the observation group was better with a lower incidence of complications than that of the control group, suggesting that TPU/NS can effectively promote patients' recovery due to the reduction of the positive rate of bacteria. Recently, studies have pointed out that one of the reasons for the outstanding antibacterial properties of nanosilver is that it has a quantum size effect, meaning that the antibacterial effect of nanosilver is related to its particle size. The smaller the particle diameter, the stronger the antibacterial effect. This is the reason why the antibacterial effect of nanosilver in the clinic is stronger than that of ordinary anionic antibacterial agents (30, 31). Finally, we compared both groups on the quality of life, and the results showed that the observation group was under a remarkably higher quality of life, indicating that optimized TPU/NS dressing after the operation can reduce the psychological and physiological trauma stress response of patients to reduce complications, shorten hospital stay, reduce the risk of readmission, and reoperation rate, thus promoting the recovery of patients as soon as possible.

However, in this study, we did not set up a blank control group using the TPU membrane alone, and prospective observation was lacking, which may offset the results. In addition, the safety of TPU/NS dressings should be evaluated by determining the liver and kidney function in diabetic patients with open fractures of lower extremities, as well as serum deposition because hepatocellular damage and nephropathy were associated with diabetes (32). Furthermore, the wound-healing rate would be assessed in the future to validate our clinical results. The above shortcomings should be improved in the follow-up study.

## Conclusion

We mixed NS into porous TPU membranes by adopting biomimetic polydopamine and then prepared a kind of wound dressing that has biocompatibility, flexibility, and antibiosis. The whole production process is simple, gentle, and environmentally friendly. TPU/NS dressing has powerful mechanical strength and great flexibility, exhibiting acceptable antibacterial activity against a variety of bacteria. We applied it to the antibacterial treatment of postoperative wounds of DM patients with joint fractures. TPU/NS dressings can lighten the postoperative pain of patients by inhibiting bacterial infection of wounds and promoting postoperative recovery of patients. Therefore, there was a tremendous potential to apply the constructed TPU/NS membrane to open fracture diabetic patients, especially those with soft tissue injury.

## Data Availability

The original contributions presented in the study are included in the article/Supplementary Material, further inquiries can be directed to the corresponding author/s.
